# The Modulation by Anesthetics and Analgesics of Respiratory Rhythm in the Nervous System

**DOI:** 10.2174/1570159X21666230810110901

**Published:** 2023-08-11

**Authors:** Xuechao Hao, Yaoxin Yang, Jin Liu, Donghang Zhang, Mengchan Ou, Bowen Ke, Tao Zhu, Cheng Zhou

**Affiliations:** 1 Department of Anesthesiology, West China Hospital of Sichuan University, Chengdu, 610041, China;; 2 Laboratory of Anesthesia and Critical Care Medicine, National-Local Joint Engineering Research Centre of Translational Medicine of Anesthesiology, West China Hospital of Sichuan University, Chengdu, 610041, China

**Keywords:** General anesthetics, analgesics, respiratory rhythm, synaptic transmission, neurotransmitter, ion channels

## Abstract

Rhythmic eupneic breathing in mammals depends on the coordinated activities of the neural system that sends cranial and spinal motor outputs to respiratory muscles. These outputs modulate lung ventilation and adjust respiratory airflow, which depends on the upper airway patency and ventilatory musculature. Anesthetics are widely used in clinical practice worldwide. In addition to clinically necessary pharmacological effects, respiratory depression is a critical side effect induced by most general anesthetics. Therefore, understanding how general anesthetics modulate the respiratory system is important for the development of safer general anesthetics. Currently used volatile anesthetics and most intravenous anesthetics induce inhibitory effects on respiratory outputs. Various general anesthetics produce differential effects on respiratory characteristics, including the respiratory rate, tidal volume, airway resistance, and ventilatory response. At the cellular and molecular levels, the mechanisms underlying anesthetic-induced breathing depression mainly include modulation of synaptic transmission of ligand-gated ionotropic receptors (*e.g*., γ-aminobutyric acid, N-methyl-D-aspartate, and nicotinic acetylcholine receptors) and ion channels (*e.g*., voltage-gated sodium, calcium, and potassium channels, two-pore domain potassium channels, and sodium leak channels), which affect neuronal firing in brainstem respiratory and peripheral chemoreceptor areas. The present review comprehensively summarizes the modulation of the respiratory system by clinically used general anesthetics, including the effects at the molecular, cellular, anatomic, and behavioral levels. Specifically, analgesics, such as opioids, which cause respiratory depression and the “opioid crisis”, are discussed. Finally, underlying strategies of respiratory stimulation that target general anesthetics and/or analgesics are summarized.

## 1 INTRODUCTION

In 1846, the successful implementation of ether anesthesia at Massachusetts General Hospital by William Morton marked the beginning of modern general anesthesia [1]. Since then, general anesthetics have become particularly important for their ability to produce various clinically required actions, mainly including hypnosis, amnesia, and immobility. In the clinical setting, general anesthesia usually consists of a combination of intravenous, volatile, and narcotic analgesics. However, many, if not all, anesthetic and/or analgesic regimens have dose-dependent or dose-independent side effects, such as interruption of normal physiological functions, suppression of the circulatory system, or influences on respiration [2]. Among them, the adverse effects on respiration have received extensive attention.

Anesthetics and narcotic analgesics have multiple sites of action within the respiratory system. These sites include the supra pontine structures involved in the volitional control of breathing, *e.g*., the respiratory brainstem neurons involved in the respiratory central pattern generator (mainly referring to the preBötzinger complex, preBötzC), efferent nerves from the brain stem, spinal motor neurons, including the medullary respiratory bulbospinal neurons (RBSNs), central and peripheral chemoreceptors, including the retrotrapezoid nucleus (RTN) and carotid body (CB), breathing-related muscles in the diaphragm, intercostal muscles, and muscles involved in upper airway patency [3, 4].

Here, we briefly summarize the current knowledge of the most important cellular and molecular targets of general anesthetics and narcotic analgesics in animal models and clinical settings. Then, we discuss their effects on respiratory control and explore potential strategies for alleviating respiratory depressant effects to facilitate the safe implementation of general anesthesia.

### 1.1 Summary of the effects of Anesthetics and Narcotic Analgesics on Breathing

Most anesthetics and analgesics can produce mild to severe respiratory depressant effects *via* the brainstem nuclei, respiratory neurons, and neuronal circuits. Respiratory control is a vital and complex behavior that is regulated by multiple internal and external factors. Therefore, it is difficult to clarify the net effects of anesthetics and analgesics on all neural structures with regard to respiration. For instance, propofol is widely used for sedation and/or hypnosis in invasive medical procedures and is considered to predominantly enhance γ-aminobutyric acid (GABA) receptors but may also inhibit glutamate release [5-7]. Although it has a rapid onset and offset, propofol also produces respiratory depression, which occurs at multiple levels, including the respiratory central pattern generator, central chemosensitive receptors, and molecular targets, such as GABA receptors, nicotinic acetylcholine receptors (nAChRs), and TASK-1 channels [8, 9], which ultimately affects the behavioral characteristics of respiratory movement. In contrast to propofol, etomidate, also a GABAergic modulator, produces less effect on respiratory depression [10, 11] and is widely used during intubation in emergency and intensive care units because of its favorable safety profile [12-14]. Interestingly, although all volatile agents induce respiratory depression, the respiratory motor system is relatively resistant to volatile anesthetics compared tointravenous general anesthetics, such as propofol in animals [15].

Narcotic analgesics, mainly opioids, are widely used to relieve surgical or cancer-related pain. Their main life-threatening side effect is respiratory depression, especially when opioids are abused or used in combination with other sedatives. Therefore, the use of opioids is limited, resulting in insufficient analgesia. The main characteristic of opioid-induced respiratory depression (OIRD) is slow and shallow breathing [16], which is mainly characterized by depression of the respiratory rate and the peak inspiratory airflow, an increase of inspiratory time, an additional pause phase before the subsequent breath [17], and a reduced hypoxic ventilatory response (HVR) and hypercapnic ventilatory response (HCVR) [18]. Respiratory depression caused by opioids is mainly mediated by the modulation of μ-opioid receptors (MORs), which are expressed on respiratory neurons in the central nervous system [16].

In brief, as one of the most vulnerable and vital physiological functions, breathing is often suppressed by most clinically used anesthetics and analgesics, including volatile anesthetics, analgesics, and most intravenous anesthetics. Although the exact mechanisms of how anesthetic/analgesics influence respiration are still being fully elucidated, it is generally known that these drugs differentially modulate respiratory-related neural nuclei and/or circuits by varied cellular and molecular targets and then suppress overall respiration to different degrees. The specific signatures of the varied anesthetics on respiration also determine their clinical application for some particular setting; therefore, understanding the exact modulatory actions of anesthetics on respiration is also clinically necessary.

## 2 SUMMARY OF THE MECHANISMS OF DRIVE AND CONTROL OF BREATHING

### 2.1 Brain Regions and Nuclei

In mature mammals, breathing is primarily controlled by neuronal activities originating in the lower brainstem [19, 20]. This neuronal activity originates from the preBötzC, where the inspiratory phase of the respiratory rhythm is produced, and the Bötzinger complex (BötzC), which generates the expiratory signals for the principal respiratory rhythm [21, 22]. Respiratory rhythm is coordinated with pattern-generating elements and relayed to premotor neurons of the rostral and caudal divisions of the ventral respiratory group (VRG) and the dorsal respiratory group [21, 23]. The neural respiratory output then drives the inspiratory and expiratory muscles, including the pharyngeal muscles and laryngeal muscles, playing an important role in the maintenance of airway patency synchronously with respiration [24-27].

The rhythmic output of respiratory centers and muscle activity comprises three phases: inspiration, in which inspiratory muscles contract; post-inspiration, in which inspiratory muscles progressively cease contraction; and active expiration, in which the expiratory muscles contract [28]. The triphasic respiratory rhythm is shaped by tonic and phasic inhibitory and excitatory inputs from the chemosensitive RTN, Kölliker-Fuse (KF) nucleus, parafacial respiratory group, post-inspiratory complex, and medial parabrachial nuclei [29-34]. Accordingly, the respiratory cycle can be divided into five components: central inspiratory activity, termination of inspiratory activity, postinspiration, active expiration, and termination of expiratory activity [19, 35]. Previous studies have indicated that respiratory rhythms or patterns can be reset. Electrical stimulation of the midbrain reticular formation and periaqueductal gray matter resulted in phase resetting and facilitation of the respiratory rhythm in cats [36]. Meza *et al.* also found that central afferent stimuli, such as scratching, delivered to the respiratory central pattern generator can reset the activity of the phrenic nerve and interneurons of the medulla oblongata in decerebrate paralyzed cats [37].

### 2.2 Respiratory Neurons

There are two types of respiratory neurons, namely, excitatory inspiratory and expiratory neurons, according to their phasic activity characteristics in the respiratory cycle [35]. Early inspiratory and augmenting inspiratory neurons depolarize and discharge in the inspiratory phase and hyperpolarize during expiration [38]. Late inspiratory or inspiratory off-switch neurons depolarize during the transition phase from inspiration to expiration and hyperpolarize during early inspiration and expiration [39, 40]. Post-inspiratory neurons depolarize only during the post-inspiration phase, with a decrementing discharge pattern, and hyperpolarize during active expiration. Augmenting expiratory neurons display augmenting depolarization during active expiration and incrementing hyperpolarization during inspiration [39, 41].

Apart from excitatory neurons in the brainstem, GABAergic inhibitory interneurons play critical roles in the interactions of interneurons that generate and modulate phasic respiratory patterns [42]. Bulbospinal, laryngeal, and pharyngeal neurons provide inhibitory inputs to modulate the discharge frequency and amplitude of the pattern generator [43-45].

### 2.3 Neural Circuits

Multiple brain regions and nuclei project to one another within the respiratory network. Yang *et al.* discovered that the preBötzC sends direct excitatory and inhibitory projections in parallel to distinct targets throughout the brain, including the middle brain, forebrain regions, and limbic regions, which generate and modulate breathing patterns [24]. A previous study reported that RTN lesions impaired CO_2_-induced arousal, carotid body (CB) ablation impaired arousal in response to hypoxia and hypercapnia, and C1 neuron ablation had no effect on arousal, indicating potential projective relationships between respiratory and arousal-related nuclei [46]. Synaptic input from astrocytes has also been demonstrated to modulate neuronal activity in the RTN [47-49].

Structures outside the brain also affect respiration. Peripheral chemosensitive neurons in the CB comprise a predominant component of the system that drives the respiratory response to hypercapnia and hypoxia [46, 50, 51]. Lung and muscle sympathetic activities are also responsive to respiration and involved in maintaining homeostasis [52, 53].

Therefore, anesthetics have multiple targets of action within the respiratory system and act in a dose-dependent or dose-independent manner. These targets include brainstem neurons that generate, shape, modulate, and integrate respiratory activity, the diaphragm, intercostal and upper airway muscles, airway smooth muscle, vagal control mechanisms, and peripheral chemoreceptors that convey afferent input to respiratory centers, such as the CB [4, 23, 54]. These mechanisms in the control of respiration are summarized in Figs. (**1** and **2**).

## 3 BEHAVIORAL CHARACTERISTICS OF RESPIRATION ARE MODULATED BY GENERAL ANESTHETICS

Breathing is one of the most vulnerable physiological indicators and is modulated by anesthesia, sleep, and exercise [55, 56]. Multiple studies have demonstrated variations in the behavioral characteristics of respiration during general anesthesia and sleep, including decreased frequency and tidal volume, reduced reactivity to hypoxia and hypercapnia, and changes in the respiratory pattern [57, 58]. Although most general anesthetics induce a sedative state similar to deep non-rapid eye movement sleep, their influence on respiration is much different from that of natural sleep [57, 59]. In addition, general anesthetics show different effects on the behavioral characteristics of respiration through varying mechanisms of action. The effects of various general anesthetics on the behavioral characteristics of respiration are summarized in Table **1**.

### 3.1 Frequency and Tidal Volume

Most intravenous general anesthetics, such as propofol, thiopental, etomidate, and ketamine, influence respiratory frequency and tidal volume. Bolus injection of propofol significantly inhibits spontaneous respiration [60, 61]. Blouin *et al.* reported that 2.5 mg/kg propofol or 4 mg/kg thiopental decreased tidal volume and increased respiratory frequency within 1 minute after injection, with the end-tidal PO_2_(PetCO_2_) maintained at 46 mmHg in volunteers [62]. Intravenous injection of clinically relevant dosages of ketamine decreased respiratory frequency and volume with respiratory pattern changes [63, 64]. Intraperitoneal administration of S-(+) ketamine at dosages of 0, 10, 100, and 200 mg/kg caused dose-dependent respiratory depression [63]. However, subjects showed better tolerance to slower administration or lower doses of intravenous agents. Injection of a 1-mg/kg bolus of propofol, followed by administration of 0.5 mg/kg every 3 minutes, resulted in subclinical respiratory depression in 42.2% of subjects, which was obviously reduced to 4.6% after the use of a bag-valve-mask apparatus [65]. Slower administration of 4 mg/kg propofol over 3 minutes preserved spontaneous respiration in 95% of pediatric patients [66]. A dose of 2 or 3 mg/kg thiopental increased respiratory frequency in decerebrate cats, with the end-tidal CO_2_ concentration controlled between 4% and 5% [67]. Nevertheless, etomidate has a better safety profile than thiopental and/or propofol and has become popular because of its ability to safely induce anesthesia while maintaining respiratory and hemodynamic stability [2, 68]. Etomidate at a dose of 0.3 mg/kg can induce hypnosis with minimal cardiorespiratory effects. However, Morgan *et al*. found that most patients experienced a brief period of hyperventilation, followed by a period of respiratory depression, and some patients even experienced apnea [69]. The incidence rates of O_2_ desaturation and respiratory depression were significantly lower under etomidate anesthesia than under propofol anesthesia [70]. The overall incidence of apnea with administration of 0.3 mg/kg etomidate was approximately 30%, and episodes lasted for 30 s on average [69]. In contrast to conventional intravenous anesthetics, dexmedetomidine, a selective α_2_ agonist, induced mild depression in the frequency of spontaneous respiration in clinical studies [71-73].

Meanwhile, almost all clinically used volatile anesthetics can suppress inspiration [3]. Volatile anesthetics increase breathing rate and decrease tidal volume and minute ventilation at 1 minimum alveolar concentration (MAC), which ultimately increases arterial PCO_2_ by 5-15 mmHg [4]. However, depression of the respiratory frequency, tidal volume, and minute ventilation occurs at higher concentrations [74]. C57BL/6J and C3H/HeJ inbred mice exposed to increased concentrations of isoflurane exhibited a progressive decrease in respiratory rate and an increase in tidal volume [75]. In C3H/HeJ mice, spontaneous ventilation was less affected during sevoflurane anesthesia than during either isoflurane or desflurane anesthesia at equivalent anesthetic potencies [76]. In goats, spontaneously breathing exhibited a dose-dependent decrease in tidal volume and ventilation under exposure to 1-2 MAC of sevoflurane, isoflurane, and halothane; among them, halothane had the greatest effect on respiratory depression [77]. However, when considering the magnitude of the PCO_2_ as an indicator, the order of depressive potency was as follows: enflurane > desflurane > isoflurane > sevoflurane > halothane [4].

Interestingly, *Lazarenko RM* and colleagues observed that isoflurane had a biphasic effect on respiratory output in rats, with an initial increase in phrenic nerve discharge (PND) frequency and a reduction in PND frequency after prolonged exposure to 1.5% and 2% isoflurane [78]. The final PND frequency returned to the initial control level when 1.5% isoflurane was administered but remained below the initial control level when 2% isoflurane was used. These results partly revealed the mechanism of breathing maintenance under isoflurane anesthesia [78].

Therefore, among the intravenous general anesthetics that are widely used in the clinical setting, propofol, thiopental and/or ketamine influence the respiratory frequency and tidal volume in a dose- and/or time-dependent manner, whereas etomidate and dexmedetomidine induce mild depression of the frequency of spontaneous respiration. For volatile anesthetics, although isoflurane has a biphasic modulating effect on frequency, nearly all volatile anesthetics produce an overall depressing effect on respiration.

### 3.2 End-tidal/Arterial PCO_2_ and Hypercapnic Ventilatory Response

In mammals, the respiratory chemoreflex regulates breathing in response to changes in brain CO_2_/H^+^ to maintain constant stability of the internal environment [30]. This phenomenon is called the hypercapnic ventilatory response (HCVR). In other words, arterial PCO_2_ is either mainly a respiratory regulator or a functional indicator of respiratory movement. Drugs that reduce CO_2_ sensitivity tend to increase the arterial PCO_2_ in subjects with spontaneous breathing; however, due to the intact chemical feedback loop formed by respiratory chemoreceptors, this effect may be partly offset and re-equilibrated [4]. In other words, the arterial PCO_2_ amplitude is influenced not only by the depressant effect of anesthetics but also by the stimulatory effects of CO_2_ on breathing [79]. Therefore, an intact regulatory mechanism may conceal residual anesthetic effects. At present, techniques to determine the ventilatory response to CO_2_ include the steady state technique and rebreathing technique [80-82]. In a steady-state study, a pneumotachograph was connected to a T-piece, one arm of which received a gas mixture (consisting of O_2_, N_2_, and CO_2_) with a flow rate of 40 L/min from mass flow controllers, by which the flow could be set individually at a desired level [80]. The computer provided control signals to the mass flow controllers so that the composition of the inspiratory gas mixture could be adjusted to keep the PetCO_2_ constant. The rebreathing technique uses a small, 4-6 L rebreathing bag filled with a gas mixture of 7% CO_2_ in 70%-80% O_2_ [82]. In this method, initiation of rebreathing close to the mixed venous CO_2_ tension leads to a large reduction in the arterial-tissue CO_2_ gradient and rapid equilibrium among the PetCO_2_, arterial PCO_2_, venous PCO_2_ and presumably brain tissue PCO_2_, assuming that the PetCO_2_ is equal to the arterial PCO_2_ [82].

Clinical respiratory responses depend on the interaction between CO_2_ and anesthetic agents in central or peripheral chemoreceptors. Propofol can directly depress respiration by blunting the chemoreceptor response to arterial PCO_2_, or it can, in turn, stimulate respiratory chemoreceptors to drive breathing through the accumulation of CO_2_ caused by respiratory depression [66, 83]. A study by Dosani *et al*. reported that a slower increase in brain propofol concentrations allows for compensatory effects, with an increase in arterial CO_2_, to drive ventilation [66]. It revealed that slow administration of propofol resulted in weaker respiratory depression. Sodium pentobarbital, thiopental or etomidate can all cause significant downward displacement of HCVR in cats or in human volunteers [62, 84, 85]. Additionally, both intravenous and/or intraperitoneal administration of ketamine can dose-dependently exhibit a significant decrease in the CO_2_ response curve slope [63, 86, 87]. Although dexmedetomidine merely produces profound hypoxemia or hypercapnia in rabbits, it still depresses the respiratory response to CO_2_ [88]. Notably, the action of anesthetics on the respiratory response to CO_2_ is different in disease states. After injection of midazolam and thiopental, the CO_2_ response curve slope decreased more profoundly in subjects suffering from chronic obstructive pulmonary disease than in healthy volunteers [89]. On the basis of these studies, such patient populations should be more closely observed because they are more vulnerable to respiratory depression.

Depressant effects on the respiratory system are dose-dependent and, as discussed above, occur at subanesthetic concentrations. Notably, the hypoxic ventilatory response (HVR) remains largely intact under subanesthetic concentrations but is entirely depressed by volatile agents at an anesthetic level [4, 84, 90]. In addition, the HCVR is more resistant than the hypoxic response, which shows a reduction by approximately two-thirds and a simultaneous increase in the apneic threshold at an anesthetic level [4, 84, 90]. This differential regulation between the HCVR and HVR might underlie the different cellular mechanisms in hypercapnic and hypoxic responses [84].

C3H/HeJ inbred mice are characterized by a blunted response to hypoxia and hypercapnia and a genetically blunted respiratory drive [87, 91]. Previous studies have demonstrated that in C3H/HeJ mice, HCVR was significantly impaired by 0.5 MAC isoflurane, sevoflurane, or desflurane and remained depressed even at the end of anesthesia recovery [76]. The depressant effects of residual isoflurane on the HCVR and HVR in C3H/HeJ mice were enhanced compared to those in wild-type mice during the recovery period [75]. Awake-intubated goats were relatively resistant to 0.5% halothane, but at 1.25%, both the HVR and HCVR were similarly and substantially reduced [92].

### 3.3 End-tidal/Arterial PO_2_ and the HVR

The HVR in humans is biphasic and consists of an initial increase in ventilation, followed by a secondary roll-off, usually designated hypoxic ventilatory decline [50, 93]. The acute isocapnic ventilatory response to hypoxia (AHR, *i.e*., the response after 3 min of hypoxia) is mediated by the CB; the secondary decline, namely, the hypoxic ventilatory decline, is mediated by CB-driven central inhibitory mechanisms [94].

Gautier *et al.* found that during an increase in PetO_2_ from 45 to 80 mmHg, sodium pentobarbital reduced the total minute ventilation by more than 50% compared to awake cats, and the expiratory and inspiratory durations were also decreased [95]. In rats, propofol acts directly on CB glomus cells to inhibit responses to hypoxia *in vitro*, which may occur *via* a novel mechanism rather than by known target receptors such as nicotinic receptors, GABAergic receptors, and calcium (Ca^2+^) or K^+^ channels [96]. In rabbits, propofol reduced the hypoxic response of the CB *in vitro* and *in vivo* through nAChRs and/or possibly K^+^ channels [8, 9].

In contrast to the HCVR, the HVR is more vulnerable to volatile anesthetics. Volatile anesthetics at clinical concentrations abolish the ventilatory response to acute isocapnic hypoxia [4]. Even under hypercapnic conditions, hypoxia did not increase ventilation but instead induced ventilatory depression by a direct inhibitory effect on the brainstem [4]. Halothane reduced the HCVR curve slope to approximately 48% compared with the control slope, while the response to hypoxia was reduced to approximately 58% of the control response [97]. A meta-analysis by Pandit confirmed that volatile anesthetics reduced the AHR by 30% 1982 with an order of inhibitory potency as follows: halothane > enflurane > sevoflurane > isoflurane > desflurane [90, 98]. In awake humans, the HVR to a moderate hypoxic stimulus (PaO_2_, 44-45 mmHg) doubled the minute ventilation but was reduced to less than one-third at 0.1 MAC halothane and entirely eliminated at 1.1 MAC halothane [99]. In addition, the depressive response to hypoxia under anesthetics is mainly modulated by the brainstem, which indicates that central chemoreceptors rather than the peripheral chemoreflex loop play a critical role in the drive to breathe under general anesthesia [100].

In brief, all volatile anesthetics may act as an inhibitor of HCVR and/or an even more potent inhibitor of HVR. Most intravenous general anesthetics reduce HCVR, while propofol and sodium pentobarbital simultaneously inhibit HVR. Therefore, at the behavioral level, general anesthetics inhibit HCVR and HVR both in human and animal studies, causing an increase in arterial PCO_2_ and a decrease in arterial PO_2_  *via* inhibition of respiratory frequency, tidal volume and minute ventilation.

### 3.4 Interaction of the Hypoxic and Hypercarbia Response

In 1974, Weiskopf and colleagues demonstrated that halothane anesthesia interfered with the interaction of hypoxia and hypercarbia in driving ventilation in spontaneously breathing dogs, which showed increased depression of the ventilatory response to CO_2_ as the PaO_2_ progressively decreased [101]. Additionally, the study suggested that halothane depressed the isocapnic HVR to a greater degree than it depressed the response to CO_2_ [101]. In anesthetized, paralyzed, vagotomized dogs with constant mechanical ventilation, Stuth *et al*. found that halothane dose-dependently (0.5-2 MAC) depressed the phrenic nerve response to a brief hypoxic stimulus but did not completely abolish it at 2 MAC. Notably, the O_2_ and CO_2_ interaction was eliminated in this configuration using an open loop configuration to eliminate all depressant effects of anesthetics distal to the phrenic nerve [102].

### 3.5 Peripheral Chemoreflex

Although the basic elements constituting the central respiratory network are located within the brainstem, structures outside the brainstem also affect respiration and maintain homeostasis, such as the CB, as a primarily peripheral chemoreceptor [23].

Peripheral chemoreflexes in humans are exquisitely sensitive to volatile anesthetics in the range of 0.1-0.2 MAC, where even memory function remains relatively intact [103]. In humans, the peripheral chemoreflex loop is completely suppressed at ≥ 1 MAC of volatile anesthetics [103]. Therefore, any changes in the CO_2_ response at these anesthetic concentrations reflect effects on the central chemoreception mechanisms, the respiratory rhythm pattern generator in the brainstem, and the transmission of inspiratory and expiratory drive to the airway and spinal cord respiratory neurons [3]. A systematic review from Pandit *et al.* also reported that volatile anesthetics at 0.75 ± 0.40 MAC depressed CB function by 24%, indicating a hypoxic response [100]. In humans, the intravenous anesthetic propofol depresses ventilatory responses to hypoxia. Animal studies suggested that this may, in part, be because of the inhibition of synaptic transmission between chemoreceptor glomus cells of the CB and the afferent carotid sinus nerve [96]. Davies *et al.* observed that 0.5-1.0% halothane in cats reduced carotid sinus nerve discharge in response to several peripheral chemoreceptor stimuli [97]. Substantial studies have demonstrated that most volatile anesthetics depress the hypoxic response of glomus cells in the CB [104-107]. Interestingly, the degree of depression with a mixture of 3% isoflurane and 1.5% halothane was less than that of 1.5% halothane alone but similar to that of 3% isoflurane alone. This phenomenon indicated that competitive interactions occur between halothane and isoflurane in the CB.

### 3.6 Upper Airway Patency

During wakefulness, airway patency is protected by pharyngeal muscle tone [108]. Similar to the effects of neuromuscular blockers, upper airway muscles are more sensitive than the diaphragm or intercostal muscles to relaxation by anesthetics, which results in loss of muscle tone and upper airway obstruction, which can be defined as negative airway pressure resulting in flow limitation or airway collapse [109, 110]. Notably, patients with a tendency for upper airway obstruction during sleep are also more vulnerable during anesthesia and sedation [110, 111].

Except for phenobarbital and ketamine, most intravenous anesthetics impair genioglossal muscle activity and upper airway patency [4, 112]. During intravenous administration of pentobarbital anesthesia, phasic genioglossus activity significantly increased, but phasic diaphragmatic activity decreased in a dose-dependent manner [113], which might be due to the inhibition of inhibitory neurons in the nucleus tractus solitarius (NTS) that project to the hypoglossal nucleus [113, 114]. However, pentobarbital still impaired respiratory genioglossus activity compared to the awake state, but the decrease was not greater than that reported during natural rapid eye movement sleep in rats [113]. Younes also found that pentobarbital increased the maximum genioglossus activity before arousal compared with placebo in rats, even during baseline breathing without CO_2_ stimulation [115]. Lorazepam and zolpidem directly increased the baseline genioglossus activity in the absence of hypercapnic stimulation. However, lorazepam and zolpidem had an inhibitory effect on genioglossus activity when applied directly to the hypoglossal motor nucleus [114]. Administration of thiopentone decreased the activity of the genioglossus, sternothyroid and sternohyoid, and scalene muscles [116]. Similarly, propofol dose-dependently decreased the phasic genioglossus activity and inspiratory duty cycle [112, 117]. Although dexmedetomidine exerted a relatively minimal effect on the ventilatory drive, it exhibited similar degrees of pharyngeal collapsibility at levels of mild to moderate sedation compared to propofol [118]. In contrast, ketamine significantly reduced the inspiratory burst frequency but increased the peak integrated inspiratory phase hypoglossal activity both *in vivo* and *in vitro* [119]. Eikermann and colleagues also demonstrated that ketamine markedly increased phasic genioglossus activity and abolished loss-of-consciousness-induced upper airway patency dysfunction in rats compared to propofol [112]. Therefore, ketamine may help to maintain upper airway patency and compensatory neuromuscular responses to upper‐airway obstruction during sedation and anesthesia [112, 117].

Eikermann and colleagues found that isoflurane increased phasic genioglossus activity and flow rate and decreased tonic genioglossus activity in vagally intact rats in a dose-dependent manner. Both the phasic and tonic genioglossus activities were markedly higher under isoflurane than under propofol [120]. The upper airway patency was also significantly decreased by volatile anesthetics at approximately 0.5 MAC [121, 122]. Compared with rats during natural behaviors, rats under urethane and halothane anesthesia exhibited an increase in the respiratory drive of transmission to the hypoglossal motor nucleus and genioglossus through non-N-methyl-D-aspartate (non-NMDA) receptors [123]. This finding indicated that anesthetics can alter the balance between receptor systems. Conventional lateral radiographs demonstrated that 3-5 mg/kg thiopentone or 3% enflurane resulted in obvious occlusion of the airway, including occlusion of the posterior pharyngeal wall of the soft palate, tongue base, and epiglottis. Occlusion occurred at all levels of the upper airway, but the tongue base did not entirely touch the posterior pharyngeal wall, resulting in complete obstruction [124]. An electromyographic study by Ochiai *et al*. revealed that halothane significantly attenuated inspiratory muscle activity in a dose-dependent manner within a wide concentration range of 1% to 3%, and the genioglossus was more sensitive than the diaphragm and intercostal muscles to halothane anesthesia [122].

### 3.7 Airway Responses

Except for thiopental, most intravenous anesthetics decrease airway responses [125, 126]. For instance, midazolam, propofol and ketamine have a direct relaxant effect on the isolated airway smooth muscle of dogs [126], and etomidate and propofol had a reduced histamine-induced contraction in isolated human airway smooth muscle [125]. Dexmedetomidine attenuated airway hyperresponsiveness and airway inflammatory responses through the TLR4/NFκB signaling pathway in ovalbumin-induced allergy model rats [127].

Bronchoconstriction and/or bronchiectasis induced by irritant anesthetics is a major concern in clinical practice, especially in patients with asthma or chronic obstructive pulmonary disease [128]. Habre *et al.* showed that isoflurane, sevoflurane, desflurane, and halothane are effective volatile agents that prevent methacholine-induced airway constriction in rats [129]. Pabelick *et al.* demonstrated that volatile anesthetics, such as halothane, isoflurane, and sevoflurane, inhibited a Ni^2+^/La^3+^-sensitive store-operated Ca^2+^ influx mechanism in porcine airway smooth muscle cells, which likely helps maintain anesthetic-induced bronchodilation [130]. Halothane and enflurane were equally effective in decreasing pulmonary resistance in asthma model dogs [131]. Meanwhile, Eilers *et al.* found that isoflurane and desflurane increased the intracellular Ca^2+^ concentration by activating the excitatory ion channel transient receptor potential-A1, which contributed to airway irritation and neurogenic bronchoconstriction in guinea pigs [128]. In practice, desflurane is regarded as a severe irritant volatile anesthetic that should be avoided for anesthesia induction, especially in children [132, 133]. Specifically, laryngospasm occurs more frequently in association with desflurane anesthesia than with isoflurane anesthesia in pediatric patients supported with laryngeal mask airways [134].

In brief, behavioral respiration under general anesthesia is modulated by general anesthetics. General anesthetics produce extremely complex modulations in every phase of breathing movement, including generation, shape and integration, resulting in regulation of frequency, tidal volume, hypercapnic or hypoxic ventilatory response and airway response. Meanwhile, to maintain an adequate oxygen supply and the stability of the internal environment under physiological or pathological conditions, the parameters of respiratory movements are inter-regulated by each other. Thus, the respiratory behaviors of individual subjects can be varied under anesthesia and significantly affected by even a small abnormality in the respiratory system. For example, breathing can be more sensitive to general anesthetics in a patient with airway obstruction, which is similar to a patient with disorders in the respiratory center.

## 4 EFFECTS OF GENERAL ANESTHETICS ON BRAINSTEM NUCLEI AND RESPIRATORY MODULATION CIRCUITS

### 4.1 General Anesthetics Modulate the Respiratory Central Pattern Generator

**Fig. (3):**
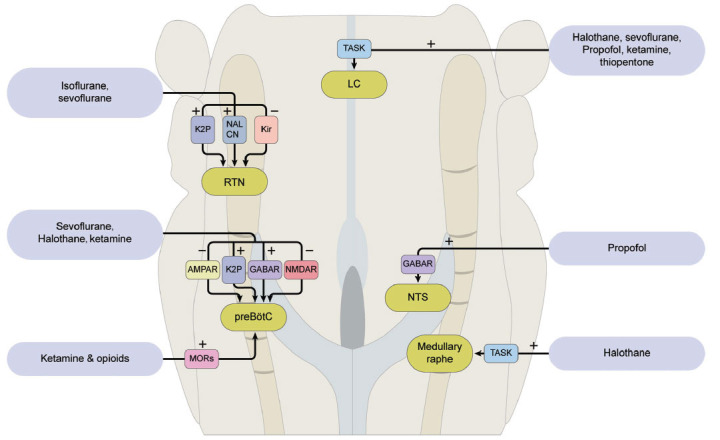
The targets of anesthetics and narcotic analgesics on breathing in the central nervous system. Anesthetics and narcotic analgesics modulate multiple nuclei within the central respiratory system (*e.g*., pre-Bötzinger complex, retrotrapezoid nucleus, nucleus tractus solitarius, locus coeruleus and medullary raphe) to affect respiratory activity. At the cellular and molecular levels, anesthetic- and/or analgesic-induced breathing depression mainly includes modulation of synaptic transmission of ligand-gated ionotropic receptors (*e.g*., γ-aminobutyric acid, N-methyl-D-aspartate and alpha-amino-3-hydroxy-5-methylisoxazole-4-propionate receptors) and ion channels (*e.g*., two-pore domain potassium channels and sodium leak channels). preBötC: pre-Bötzinger complex; RTN: retrotrapezoid nucleus; LC: locus coeruleus; NTS: nucleus tractus solitarius; GABAR: γ-aminobutyric acid receptor; AMPAR: alpha-amino-3-hydroxy-5-methylisoxazole-4-propionate receptor; NMDAR: N-methyl-D-aspartate receptor; K_2P_: two-pore domain potassium channels; MORs: μ-opioid receptors; NALCN: leak sodium channel; Kir: inward rectifier potassium channels; TASK: TWIK-related acid-sensitive K^+^ channels.

Rhythmic breathing movement is mainly derived from the respiratory central pattern generator. The preBötzC network, a spatially localized and functionally specialized region of the medulla containing a heterogeneous excitatory network with autorhythmic properties, is the substrate for inspiratory rhythm generation and the source of rhythmic excitatory drive to premotor transmission circuits in the brainstem respiratory network [135]. Kuribayashi *et al.* observed that sevoflurane caused a synchronous decrease in the burst rates of inspiratory neurons in the rostral ventrolateral medulla and of fictive inspiration recorded from ventral cervical roots in *in vitro* preparations from newborn rats, which implies that preBötzC is responsible for the depressant effect of sevoflurane on the respiratory rhythm [136]. PreBötzC contains a variety of ion channels, including not only ligand-gated ion channels that mediate fast synaptic transmission by alpha-amino-3-hydroxy-5-methylisoxazole-4-propionate (AMPA), NMDA, and GABA but also voltage-dependent ion channels that regulate neuronal excitability. The molecular targets of various general anesthetics on respiration in the central nervous system are summarized in (Table **2** and Fig. **3**).

#### 4.1.1 Two-pore Domain Potassium (K_2P_) Channels

The K_2P_ channels consist of two pore-forming regions flanked by four membrane-spanning domains and are responsible for baseline or leak conductance. The K^+^-dominated leak channel plays a fundamental role in the generation and control of preBötzC rhythmic activity *in vitro*, which is mainly mediated by TWIK-related acid-sensitive K^+^ (TASK)-like channels [137]. Volatile anesthetics activate TASK-like conductance to control preBötzC rhythmic activity and suppress rhythmic breathing [137-139]. A study by Koizumi *et al.* also found that 1-2% halothane-activated TASK-like current in preBötzC pacemaker neurons, which may be associated with depressed respiratory output in brainstem slices and is sufficient to account for respiratory depression [137].

#### 4.1.2 Glutamatergic Receptors

The most commonly used general anesthetics, isoflurane, ether, and ketamine, mainly act on the NMDA and AMPA receptor complex *in vitro* [140]. Excitatory transmission mediated by AMPA or NMDA receptors is critical for respiratory rhythm generation [141]. AMPA receptor activation and modulation by phosphorylation are crucial for rhythm generation within preBötzC [142]. Injection of AMPA at the rostral rhombencephalon increased the rate of fast respiratory rhythm but decreased the frequency of slow breathing patterns [143]. Excitatory inputs from CB chemoreceptors to expiratory bulbospinal neurons were mediated by NMDA receptors in dog expiratory neurons, and these inputs were reduced by AP5 [144]. Krolo *et al.* found that two-thirds of the excitatory drive to inspiratory neurons within the VRG is tonic and mediated by NMDA receptors [145]. Therefore, an interaction may occur between anesthetics and AMPA and/or NMDA receptor modulators in the respiratory system. Shimazu *et al*. found a respiratory depressant effect of AMPA from 1.5% halothane and/or NMDA receptor antagonists in rats [146]. Respiratory parameters were also mildly suppressed by 1.5% halothane anesthesia alone. With combined administration of AMPA or NMDA receptor antagonists, 1.5% halothane led to a significantly suppressed respiratory rate and decreased respiratory minute volume in rats [146]. This result suggested that halothane may act by modulating AMPA and NMDA receptors in respiratory circuits. In rats, the combination of NMDA receptor antagonists, such as ketamine, with morphine or other opioids results in increased respiratory depression compared to that produced by opioids alone [147]. This result implies that NMDA receptors are involved in respiratory inhibition under ketamine anesthesia.

#### 4.1.3 GABA Receptors

*In vivo* studies with microinjected agonists and antagonists reported that GABA_A_ receptors play important roles in the phasic generation of reciprocal tonic activation of medullary respiratory premotor neurons and respiratory pattern generators [148, 149]. Antagonizing GABA_A_ receptors with bicuculline amplified the discharge frequency of inspiratory and expiratory premotor neurons during their normally active phase in a dose-dependent manner without changing the silencing effect. This evidence supports the gain-modulating effect of GABAergic input on respiratory control [29, 45]. In contrast to bicuculline-sensitive inhibitory gain modulation, the silent-phase inhibition of RBSNs is mediated by pharmacologically distinct, picrotoxin-sensitive GABA_A_ receptors [19, 77].

Sevoflurane had depressant effects on the parafacial respiratory group pre-inspiratory neurons and the preBötzC inspiratory neurons, with the former inhibited by GABA_A_ergic and glycinergic receptors and the latter depressed *via* GABA_A_ergic but not *via* glycinergic inputs [150]. Meanwhile, sevofluran exhibited stimulant effects at the brainstem level, presumably in the rate-generating regions [150], which may contribute to an increase in respiratory frequency induced by sevoflurane in humans [151], goats [77], decerebrated cats [152], and decerebrated dogs [153].

#### 4.1.4 Glycine Receptors

Glycine acts as an important inhibitory neurotransmitter in the brainstem and spinal cord to modulate respiration [126]. Dutschmann *et al.* demonstrated that during the early lifespan in rats, glycine receptors coordinate the activity of cranial and spinal motor inspiratory and post-inspiratory neurons and are essential for the formation of normal breathing patterns [32]. The role of the glycine receptor in the silent-phase inhibition of BSNs has also been studied [3]. The silent expiratory phase is produced by phasic inhibition of tonic activity, and ~80% of this inhibition is mediated by GABA_A_ receptors and ~20% by glycine receptors [145]. Although it is unknown whether glycine contributes to the respiratory depressant effects of volatile and/or intravenous anesthetics, glycine receptors are still another potential target for anesthetics.

#### 4.1.5 μ-Opioid Receptors

The endogenous opioid system is vital to the physiological function of breathing control. Mice that lacked MORs displayed an approximately 10% greater level of ventilation than mice with MORs because of the increased breathing frequency [94]. However, 1% sevoflurane induced similar reductions in resting ventilation and the slope of the HCVR in MOR^+/+^ and MOR^-/-^ mice as measured using whole-body plethysmography [154], indicating that sevoflurane has little effect on MORs. S-(+)-ketamine showed greater dose-dependent depression of respiration in wild-type mice than in MOR knockout mice [63]. In dogs, the respiratory depressant effect of S-(+)-ketamine was reversed by selective μ and δ receptor antagonists [155]. These results suggested that the respiratory depression induced by ketamine may partly be mediated *via* opioid receptors.

### 4.2 General Anesthetics Modulate Central Chemoreceptors

#### 4.2.1 Retrotrapezoid Nucleus

As an important respiratory regulation center, the RTN region is highly sensitive to changes in CO_2_/H^+^, which are responsible for integrating feedback and feedforward information and transmitting it to the central rhythm generator. The RTN has not only intrinsic CO_2_/H^+^ sensitivity but also receives paracrine signals from astrocytes in the local area to regulate respiratory movement.

##### 4.2.1.1 K_2P_ and NALCN Channels

Multiple K^+^ channels are expressed in the respiratory system. Lazarenko and colleagues observed that 1.5% and 2% isoflurane increased the firing rate of RTN chemosensitive neurons independent of CO_2_ levels in rats *via* inhibition of THIK-1-like (tandem pore domain halothane inhibited potassium channel 1) channels. The leak sodium channel (NALCN) is a voltage-independent leak channel that is widely expressed throughout the central nervous system [156, 157] and is involved in maintaining and regulating the respiratory rhythm [78, 156, 158]. We recently found that clinically relevant concentrations of volatile anesthetics, such as sevoflurane and isoflurane, but not propofol, increased the activity of Phox2b neurons in the RTN by enhancing NALCN conductance [15]. This finding revealed that the NALCN channel is an important determinant in maintaining respiratory output during exposure to volatile anesthetics. Therefore, volatile anesthetics do not abolish spontaneous breathing, partly because of the activation of central chemosensitive neurons in the RTN *via* inhibition of THIK-1 channels [78] and activation of NALCN channels. Nevertheless, the combined effect of all volatile anesthetics is a reduction in ventilatory CO_2_ sensitivity [78].

##### 4.2.1.2 Inward Rectifier Potassium Channels

Inward rectifier potassium channels (Kir) contribute to maintaining the resting membrane potential, play an important role in forming the membrane potential, and modulate muscle tone in certain types of smooth muscles, including in human bronchial smooth muscle cells [159]. Kir4.1/5.1, which is co-expressed in astrocytes of brainstem cardio-respiratory nuclei, can detect PCO_2_ changes in either hypercapnic or hypocapnic conditions. It is likely that heteromeric Kir4.1/5.1 contributes to CO_2_/pH sensitivity in these neurons [160] and is important for functional central and peripheral respiratory chemosensitivity [161]. Our previous study demonstrated that isoflurane inhibits heteromeric Kir4.1/5.1 channels in HEK293T cells and Kir4.1/5.1-like conductance in astrocytes in the RTN; thus, these channels play a role in spontaneous respiratory maintenance [162].

#### 4.2.2 Locus Coeruleus and Medullary Raphe

The locus coeruleus (LC) mainly regulates arousal and analgesia, participates in the development of the respiratory network, and can modulate the respiratory rhythm. TASK channels are also expressed in raphe serotonergic neurons and LC neurons, conferring pH-sensitive and anesthetic-sensitive K^+^ conductance [163, 164]. Washburn *et al.* suggested that modulation of TASK channels by anesthetics may contribute to the clinical effects of volatile anesthetics mediated by serotonergic raphe neurons [164]. Mechanistically, the reduction in CO_2_ sensitivity by volatile anesthetics may be related to stimulation of TASK-like currents in brainstem motor neurons or pH-sensitive neurons in the medullary raphe and LC [138, 163]. These data suggested that TASK-like channels may be involved in the depressant effects of anesthetics on respiration.

#### 4.2.3 Nucleus Tractus Solitarius

Propofol (> 3 μM) increased the frequency of spontaneous excitatory postsynaptic currents and evoked glutamate release onto NTS neurons by GABA_A_ receptor-mediated depolarization of the presynaptic excitatory terminals [165, 166].

### 4.3 Peripheral Chemoreceptors

Under normal conditions, hypoxemia evokes hyperventilation. However, this protective HVR is suppressed by most, but not all, volatile and intravenous anesthetics, even at very low levels [167]. Human CB contains TASK-1 channels, GABA_A_ receptors, and nAChRs, all of which are known targets of volatile anesthetics [167]. CB Type I cells contain TASK-1 channels that participate in pH sensing and are stimulated by halothane and isoflurane in the same order of potency as that observed in the reduction of AHR in humans [106, 168, 169].

Of note, both volatile anesthetics and intravenous anesthetics at clinically relevant concentrations inhibit various subtypes of voltage-gated calcium, sodium and potassium channels, which suppress synaptic neurotransmission [170, 171]. However, it is still unclear how the effects of general anesthetics on these ion channels contribute to their respiratory depressant effect. Currently, benefiting from novel theoretical research works and/or simulation approaches, such as high-resolution measurements and molecular dynamics simulations, general anesthetics were found to directly bind with voltage-gated ion channels [172, 173]. In CB, voltage-gated K^+^ channels (K_v_), including K_v_1.2, K_v_1.5, K_v_2.1, K_v_3.1, K_v_3.3, K_v_4.2 and K_v_9.3, are reversibly blocked by hypoxia, which results in depolarization of O_2_-sensitive cells [174, 175]. For the voltage-gated potassium channel K_v_1.2, Stock and colleagues illustrated that sevoflurane could conformation-dependently bind to multiple saturable sites of K_v_1.2, even at low concentration *via* a combined docking and free-energy perturbation approach [172, 173]. As K_v_1.2 in O_2_-sensitive CB cells responds to hypoxia, sevoflurane may regulate the HVR by modulating K_v_1.2.

### 4.4 Astrocytes

Astrocytes of the brainstem chemoreceptor areas are highly chemosensitive [176, 177]. Central respiratory chemosensitivity is an essential mechanism that, *via* regulation of breathing, maintains constant levels of blood and brain PCO_2_/pH. Brainstem astrocytes detect physiological changes in pH, activate neurons of the neighboring respiratory network, and contribute to the development of adaptive respiratory responses to increases in blood and brain PCO_2_/H^+^ levels [178, 179]. Blockade of vesicular release in preBötzC astrocytes in rats reduces the resting breathing rate and frequency of periodic sighs, decreases rhythm variability, impairs respiratory responses to hypoxia and hypercapnia, and dramatically reduces exercise capacity [180].

Astrocytes respond to physiological decreases in pH with vigorous elevations in intracellular Ca^2+^ and the release of ATP. ATP propagates astrocytic Ca^2+^ excitation, activates chemoreceptor neurons, and induces adaptive increases in breathing. Mimicking pH-evoked Ca^2+^ responses by optogenetic stimulation of astrocytes expressing channelrhodopsin-2 activates chemoreceptor neurons *via* ATP-dependent mechanisms and triggers robust respiratory responses *in vivo* [181]. In pH-sensitive astrocytes, acidification activates the electrogenic Na^+^/HCO_3_^−^ cotransporter NBCe1, which transports Na^+^ inside the cell. The increasing intracellular Na^+^ concentration causes the Na^+^/Ca^2+^ exchanger to operate in a reverse mode, leading to Ca^2+^ entry, followed by activation of downstream signaling pathways [182].

## 5 EFFECT ON THE EXCITABILITY AND NEUROTRANSMISSION OF RESPIRATORY NEURONS AND CENTRAL RESPIRATORY-RELATED STRUCTURES

The respiratory rhythm originates from the combined action of two components, a rhythm generator and a pattern generator. The former organizes the switching process between the respiratory phases, and the latter shapes the neuron activity pattern [35]. The respiratory movement mainly depends on the rhythmic discharge of respiratory-related neurons. In addition to chemical neurotransmission, intrinsic membrane properties, such as voltage-dependent or voltage-independent currents, are also essential in regulating the discharge pattern of each type of respiratory neuron [35].

Overall neurotransmission depends on the amount of transmitter released and the responsiveness of the postsynaptic receptors. The effect of general anesthetics on neuronal activity is caused by the combined effects of pre- and postsynaptic excitatory and inhibitory synaptic transmission [35]. The total excitatory drive of RBSNs is regulated by excitatory inputs *via* NMDA and/or AMPA receptors [3] and tonic inhibitory inputs *via* GABA_A_ receptors, which reduce the magnitude of the discharge pattern rather than alter the time course of the pattern [183] and can be antagonized by bicuculline [149]. The medullary RBSNs are not rhythmogenic *per se*, but phasic inhibition that produces the silent phase is mediated by GABA_A_ receptors, which can be antagonized with picrotoxin rather than bicuculline [149] and glycine receptors [184].

*In vivo*, decerebrate animal models are a commonly used paradigm to investigate the effects of general anesthetics on neurotransmission to respiratory motor neurons. Decerebration removes excitatory drive inputs from the forebrain and midbrain to the respiratory pattern generator; therefore, most decerebrate dogs develop apnea at low concentrations of anesthetics compared to those in neuraxis-intact subjects [3]. Stuth *et al*. demonstrated that 1 MAC sevoflurane enhanced the overall inhibition of inspiratory neurons by 31%, while overall excitation was reduced by 9% compared to the awake state [3]. Sevoflurane and halothane similarly depressed the overall excitatory drive to inspiratory and expiratory neurons by approximately 20%. Meanwhile, halothane, but not sevoflurane, depressed postsynaptic AMPA and NMDA receptor function only in inspiratory neurons [3]. These results may indicate that the glutamate receptors between inspiratory and expiratory neurons consist of different combinations of subunits, by which various general anesthetics may differentially regulate inspiratory and expiratory neurons.

### 5.1 Inspiratory Premotor Neurons

Inspiratory neurons located in the caudal ventral medulla are premotor neurons that drive motor neurons that innervate pump muscles, such as the diaphragm and external intercostal muscles [185]. Excitatory drive to inspiratory neurons is mainly mediated by NMDA and APMA receptors and is modulated by inhibitory GABA_A_ergic input.

By picoinjection of AMPA, NMDA, and bicuculline, Stucke *et al*. found that 1 MAC sevoflurane depressed the spontaneous activity of inspiratory premotor neurons, decreased overall glutamatergic excitation, and enhanced overall GABA_A_ergic inhibition [186]. However, the postsynaptic responses to exogenous AMPA and NMDA were not altered by sevoflurane [186]. Meanwhile, in a decerebrate dog model, 1 MAC halothane depressed the spontaneous activity of inspiratory premotor neurons, decreased overall excitatory drive, and increased postsynaptic GABA_A_ receptor function [187]. Paradoxically, Stucke *et al*. also reported that 1 MAC halothane depressed the spontaneous activity of inspiratory neurons and the postsynaptic responses to exogenous AMPA and NMDA, all without changing overall GABA_A_ergic inhibition [185]. The spontaneous activity of inspiratory neurons was more likely to be altered by sevoflurane than by halothane, which was consistent with the increased depression of phrenic nerve activity caused by 1 MAC sevoflurane compared to 1 MAC halothane [153].

### 5.2 Expiratory Premotor Neurons

Systematic studies on expiratory premotor neurons in the caudal VRG revealed that 1 MAC halothane reduced neuronal activity by reducing presynaptic glutamatergic excitatory drive and increasing overall inhibition. However, the postsynaptic responses of neurons to exogenous NMDA were not significantly depressed [153]. Moreover, postsynaptic GABA_A_ receptor function was enhanced by 65% and 74% by 1 MAC halothane and sevoflurane, respectively [188].

The discrepancy in sensitivity to general anesthetics between inspiratory and expiratory neurons might be associated with different subunit combinations. Ireland *et al.* found that AMPA receptors involved in the generation of the inspiratory rhythm and the transmission of this inspiratory drive to motor neurons are differentially sensitive to UBP-302, which antagonizes GluR5 subunit-containing kainate receptors at ≤ 10 μM but antagonizes other kainate and AMPA receptors at ≥ 100 μM [189] This result suggests differential expression of non-NMDA/AMPA glutamate receptor subtypes within inspiratory rhythm-generating networks. Robinson *et al.* mapped respiratory premotor and motor neurons using combined immunohistochemistry and retrograde labeling in adult rats. Their results showed that immunoreactivity for AMPA receptor subunits was distributed throughout the soma and proximal dendrites, NMDAR1 subunit immunolabeling was localized in the soma, and GluR5-7 subunit immunolabeling was confined largely to dendrites [190]. The difference in sensitivity of various glutamate receptor subunits to general anesthetics has been demonstrated by previous studies [191, 192].

It is generally speculated that extrasynaptic GABA_A_ receptors mediate tonic currents, which are thought to play a major role in the refinement of neuronal firing pattern, whereas GABA receptors that participate in direct synaptic transmission mediate phasic inhibitory currents [193, 194]. Both intravenous and volatile anesthetics enhance the overall GABAergic inhibition or postsynaptic GABA_A_ receptor function in inspiratory and expiratory neurons [185-187, 194]. However, the effects of anesthetics on overall inhibition are not equal to the enhancement in postsynaptic GABA_A_ receptor function. A previous study on neuraxis-intact dogs showed depression of overall inhibition by increasing the concentration of halothane from 1 to 2 MAC [195]. Greater depression *via* an increased anesthetic dose decreased presynaptic inhibitory input, which might offset the effect of enhanced postsynaptic GABA_A_ receptor function. Interestingly, this dual effect of general anesthetics on neuron activity was also demonstrated by previous and recent studies [196, 197].

Using anesthetized, vagotomized, paralyzed, and mechanically ventilated dogs, Stuth *et al*. showed that the depressant effect of halothane on bulbospinal expiratory neurons was caused by inhibition of NMDA receptor-mediated excitatory input into these neurons rather than enhancement of GABA_A_ receptor-mediated inhibitory input [195, 198]. Vanini *et al.* demonstrated that isoflurane decreased GABA levels in the pontine reticular formation and contributed to a decrease in respiratory rate and skeletal muscle activity while inhibiting GABA uptake with nipecotic acid reversed this effect [199].

Studies on synaptosomes have shown that volatile anesthetics can cause greater depression of glutamate release than GABA release [200]. Housely and Sinclair [201] demonstrated that afferent nerve fibers carry peripheral chemoreceptor impulses to respiratory centers and cause the release of the excitatory amino acid glutamate. Inhibition of glutamate receptors by the NMDA antagonist MK801 reduces the magnitude of the hyperventilatory response to hypoxia. In the acute ventilatory response to hypoxia, afferent impulses from the CB reach the NTS and the ventral medullary surface to release glutamate, which stimulates ventilation. During hypoxia, the GABA concentration also increases in the brain. This primarily occurs because of the conversion of glutamate to GABA, facilitated by the enzyme glutamic acid decarboxylase, which is a primary anaerobic enzyme in the brain. The final ventilatory drive depends on the interaction between these excitatory and inhibitory amino acids, and over time, the ventilatory output is increased or decreased [202].

### 5.3 Depression of Hypoglossal and Facial Motor Neurons

Neurotransmitters were found to increase the excitability of mammalian hypoglossal motor neurons by inhibiting TASK channels and mediating activation of the hyperpolarization-activated cationic current I(h), while anesthetics decreased excitability by activating a TASK-like current and inducing a hyperpolarizing shift in I(h) activation [203]. Halothane also inhibited I(h), which primarily occurred because of a decrease in the absolute amount of current, although halothane also caused a small, but statistically significant shift in the voltage dependence of I(h) activation [204]. In all hypoglossal motor neurons, halothane and sevoflurane induced membrane hyperpolarization by activating TASK-1 channels at clinically relevant anesthetic levels [163]. In rat brainstem motor neurons, halothane activated TASK-1 channels, induced hyperpolarization of the membrane, and suppressed action potential discharge [163, 203]. Washburn revealed that TASK-1 and TASK-3 channel mRNA was present in all hypoglossal and facial motor neurons and in most small and large NK1R (neurokinin 1 receptor-immunoreactive neurons (> 90%) of the VRG, as well as in all inspiratory-augmenting bulbospinal neurons of the rostral VRG [205].

Although serotonin (5-HT) is an agonist of TASK channels and provides an important excitatory drive to hypoglossal motor neurons, a previous study showed a weak influence of 5-HT on the subanesthetic concentration of anesthetic-induced depression with regard to hypoglossal motor neuron activity, indicating a minor role of TASK channels in this depressive effect [206]. Furthermore, Sirois *et al.* investigated the interactions of halothane and the neurotransmitters 5-HT and noradrenaline in TASK channels [203] and found that halothane and neurotransmitters have opposite effects on TASK channels and hyperpolarization-activated cyclic nucleotide-gated channels. In *in vitro* preparations, activation of TASK-1 channels by halothane was concentration-dependent and was increased by approximately 10% by 0.1 mM halothane (0.4 MAC); a maximal increase of approximately 60% was observed at supraclinical concentrations (0.9 mM halothane, > 3 MAC) in rats [163]. Interestingly, in hypoglossal motor neurons in a neonatal rat brainstem slice preparation, Sirois *et al.* found that the outward K^+^ current induced by 0.75% of halothane was less than that induced by an equivalent anesthetic concentration of sevoflurane. Therefore, sevoflurane may have a greater effect on TASK-1 channels than halothane [204].

### 5.4 Relaxation of Airway Smooth Muscle

In airway smooth muscle, homeostasis of the intracellular basal Ca^2+^ concentration is important to maintain proper airway patency [207, 208]. Based on their electrical and pharmacological properties, voltage-gated Ca^2+^ channels are classified into four types: T, L, N, and P [209]. Membrane Ca^2+^ channels, mainly including two types of voltage-dependent Ca^2+^ channels (L-type and T-type) and transient receptor potential canonical 3, control Ca^2+^ influx to maintain basal Ca^2+^ concentrations [210]. Hall *et al*. suggested that P-type Ca^2+^ channels are relatively insensitive to clinical concentrations of anesthetics [209]. Yamakage *et al*. reported that halothane, isoflurane and sevoflurane significantly inhibited macroscopic voltage-activated Ca^2+^ currents in porcine tracheal smooth muscle cells, which might contribute to the relaxation of airway smooth muscle [211]. In studies using hippocampal pyramidal neurons, isoflurane inhibited T-, L-, N-, and possibly P-type Ca^2+^ peak currents by 50% at approximately 2.6 MAC [212]. However, it is currently unclear whether this is relevant to its respiratory depressant effect. Voltage-gated Na^+^ channels are another target of inhaled anesthetics at clinical concentrations [213]. Na_v_1.4, a voltage-gated Na^+^ channel, which is encoded by the SCN4A gene, is highly expressed in skeletal muscle, including skeletal respiratory muscles [214]. SCN4A variants cause neonatal Na^+^ channel myotonia with respiratory failure because of severe apnea and thorax rigidity [215]. Although the roles of Na^+^ channels in the effects of general anesthetics and respiration are unclear, inhibition of Na_v_1.4 channels could explain some of the anesthetic inhibition of respiration [216]. Multiple K^+^ channels are expressed in the respiratory epithelium lining airways and alveoli, and the main function of K^+^ channels is to control membrane potential and maintain the driving force for transepithelial ion and liquid transport [217].

## 6 NARCOTIC ANALGESICS MODULATE RESPIRATION

The respiratory control system is highly vulnerable to exogenous opioid analgesics. Additionally, the neural substrates that contribute to OIRD pathogenesis are present at specific sites in the central and peripheral nervous system, mainly including the preBötzC, parabrachial complex (PBC), and CB [218]. Respiratory depression caused by opioids is mainly mediated by the modulation of MORs, which are widely expressed in respiratory neurons in the central nervous system [16].

Montandon and Horner proposed that the preBötzC plays a key role in OIRD because localized injection of opioids results in obvious respiratory depression. Additionally, localized naloxone administration or genetic elimination of MORs reversed the decrease in breathing after systemic administration of opioids [17, 219, 220]. For instance, continuous local unilateral application of the MOR agonist DAMGO ([D-Ala^2^, N-Me-Phe^4^, Gly^5^-ol]-enkephalin) or fentanyl into the preBötzC in adult rats caused the sustained slowing of the respiratory rate, increased respiratory rate variability, and suppressed genioglossus activity, but not diaphragm muscle activation; all of these results were reversed by the MOR antagonist naloxone [219]. The PBC includes the lateral parabrachial, medial parabrachial, and KF nuclei, which modulate breathing in response to CO_2_/O_2_ imbalance and noxious stimuli. Local injection of opioids into the parabrachial nuclei also slows breathing, which is predominantly driven by MORs of glutamatergic neurons [17, 221]. Furthermore, Bachmutsky *et al*. demonstrated that activation of MORs in the preBötzC and/or parabrachial nuclei accounts for OIRD using genetic approaches in awake mice. Meanwhile, bilateral microinjection of naloxone into the parabrachial nuclei substantially reversed respiratory depression induced by clinically relevant concentrations of remifentanil in decerebrate rabbits, whereas injection into the preBötzC did not have this effect [218]. Interestingly, the deletion of Oprm1 in the parabrachial complex did not affect breathing after saline injection, suggesting that in this context, opioids do not exert an endogenous effect [17]. The lateral parabrachial nucleus expresses MORs (PBL^Oprm1^ neurons) and is involved in OIRD pathogenesis. Chemogenetic inactivation of PBL^Oprm1^ neurons mimics OIRD in mice, whereas chemogenetic activation of these neurons following morphine injection rescues respiratory rhythms to baseline levels [222].

Opioids may have dose-dependent and region-specific effects on respiratory control. At a low dose of morphine, the deletion of MORs from KF and preBötzC neurons attenuated respiratory depression. However, at high doses of morphine, only silencing MORs in KF neurons can counter the respiratory depressant effect [223].

At the molecular level, Shijia Liu *et al.* found that several excitatory G protein-coupled receptors, such as 5-HT receptor 2A, cholecystokinin A receptor, and tachykinin receptor 1, were expressed by PBL^Oprm1^ neurons and that agonists of these receptors restored breathing rates in mice experiencing OIRD [222]. Serotonin 4a (5-HT4a) receptors are strongly expressed in the preBötzC, and their selective activation protects spontaneous respiratory activity. Stimulation of 5-HT4a receptors by a pharmacologic approach in rats could effectively counteract fentanyl-induced respiratory depression and reestablish a stable respiratory rhythm without compromising the antinociceptive potency of fentanyl [224]. NK1R-expressing preBötzC neurons are essential for stable breathing and generating the respiratory rhythm [225, 226]. Electrophysiological recordings showed that NK1R-expressing preBötzC neurons are opioid sensitive and mediate opioid-induced respiratory rate depression [219].

In contrast to the inhibitory effect of opioids on central respiratory rhythm pacemakers and central chemoreceptors, morphine at analgesic doses did not weaken CB function. Baby *et al*. found that compared with sham-operated Sprague-Dawley rats, morphine-induced suppression of the HVR and HCVR was substantially increased in bilateral carotid sinus nerve transected rats. These results suggested that morphine did not compromise CB function and that CB may defend against OIRD [18]. The molecular targets of analgesics on breathing are summarized in Fig. (**2**).

## 7 POTENTIAL STIMULATION STRATEGIES FOR RESPIRATORY DEPRESSION

General anesthetics and opioid analgesics can produce respiratory impairment of molecular targets at the cellular level and in neural circuits, which greatly compromises the safety of general anesthesia and opioid use. Therefore, strategies to reverse or relieve the depressant effect of anesthetics and/or analgesic agents are a fundamental necessity. Emphasis should be given to finding potential targets to modify and safely implement anesthetics and analgesics without losing efficacy. Probable strategies include enhancement of respiratory target activity and reduction or elimination of inhibitory targets. The potential stimulation targets to relieve respiratory depression of general anesthetics and/or analgesics are summarized in Table **3**.

### 7.1 Approaches to Relieving General Anesthetic-induced Respiratory Depression

Previous studies have shown that volatile anesthetics, including isoflurane and sevoflurane, enhanced NALCN-like conductance and/or inhibited THIK-1-like conductance in RTN Phox2b neurons and modulated respiratory maintenance [15, 78]. In addition, isoflurane inhibits Kir4.1/5.1-like conductance in astrocytes of the RTN and plays a role in spontaneous respiratory maintenance under general anesthesia [162]. Administration of BK_Ca_, TASK-1, and/or TASK-3 antagonists in CB glomus cells has been associated with an increase in respiratory drive and minute ventilation and reversal of anesthetic (isoflurane/propofol)-induced respiratory depression [227, 228]. A previous study showed that a 1-mg/kg bolus dose of doxapram produced modest respiratory stimulation following total intravenous anesthesia with propofol and remifentanil in perioperative patients [227]. All of the above may be potential molecular candidates to explore novel general anesthetic agents.

### 7.2 Approaches to Averting OIRD

In particular, OIRD accounts for the current global opioid crisis and results in inadequate pain relief. This situation has put forward the need to develop novel therapeutics that provide equivalent antinociception without depressing breathing movement. However, presently used reversal agents, such as naloxone, a nonselective opioid receptor antagonist, rapidly reverse opioid-induced respiratory depression at the expense of loss of analgesic effects and are usually associated with deleterious disadvantages, such as cardiopulmonary arrest [229]. Therefore, alternative candidates are promising interventions for mitigating OIRD and decreasing the probability of opioid fatalities.

These strategies include specific silencing of MORs in the respiratory control network (preBötzC and/or parabrachial nuclei), activation of G-protein coupled receptors, including 5-HT 2A receptors, 5-HT4a receptors, cholecystokinin A receptors, and tachykinin receptor 1 in PBL^Oprm1^ neurons [222] or preBötzC interneurons [224], activation of AMPA receptors in respiratory centers [229], and/or blocking of Ca^2+^-activated K^+^ channels to increase the excitability of CB cells [230] and thus modulate the respiratory network.

A previous study showed that 5-HT receptors are abundantly expressed in the preBötzC and KF nuclei and their selective activation enhances respiratory neuron activity and reduces respiratory rhythm variability [16, 224, 231]. Treatment with a 5-HT4a receptor agonist relieved fentanyl-induced respiratory depression and re-established a stable respiratory rhythm without loss of antinociception [224]. Administration of a 5-HT1a receptor agonist prevented morphine- and remifentanil-induced respiratory depression without compromising the antinociceptive effects [232] and even prolonged remifentanil-induced analgesia [233]. Injection of a 5-HT2a receptor agonist into the PBL of anesthetized mice after inducing OIRD with morphine increased the respiratory rate [222]. Background K^+^ channels in the CB [234] and brainstem [78] are important regulators of ventilation and are stimulated by hypoxia and acid. Blocking these channels increases CB signaling, phrenic nerve activity, and respiratory drive. Currently, caffeine, doxapram, and almitrine are common respiratory stimulants used clinically as breathing control modulators. These agents mainly act by modulating the CB by blocking O_2_-sensitive K^+^ channel-like Ca^2+^-dependent K_Ca_1.1 (BK_Ca_), TASK-1, and/or TASK-3 channels. Unlike opioid receptor antagonists, GAL-021 is being developed as a novel nonopioid respiratory stimulant to preserve respiratory drive and protect patients from respiratory impairment caused by opioids or other modalities and to freely reverse or compromise opioid analgesic efficacy. GAL-021 produced respiratory stimulatory effects during the alfentanil-induced respiratory depression, but it had no impact on sedation, analgesia, or hemodynamics in volunteers [228]. Administration of GAL-021 to rats or non-human primates stimulated respiration in a dose-dependent manner, including increasing tidal volume, respiratory frequency, and minute ventilation in conscious subjects, and reversed opioid (morphine/fentanyl) and benzodiazepine (midazolam)-induced respiratory depression. BK_Ca_ channels may be potential substrates for the reversal of opioid- and nonopioid-induced respiratory depression. AMPA receptors are present in key central nervous system centers of the respiratory drive, such as the preBötzC, as well as sites outside the preBötzC. Activation or inhibition of AMPA receptors in respiratory control systems results in respiratory stimulation or inhibition, respectively [235, 236]. For instance, an ampakine, CX717, has been demonstrated to increase respiratory volume and frequency in both animals and humans, but only under hypoventilation [237].

## CONCLUSION

The well-known cellular and/or molecular targets related to respiratory function are inter-regulatory. The modulatory effect of general anesthetics on respiration is, therefore, elaborate and sophisticated. In brief, current studies have confirmed that the negative modulations of general anesthetics and analgesics on breathing movement are mainly mediated by the activation of K^+^ channels and/or inhibition of Na^+^ channels and Ca^2+^ channels, which lead to suppression of the excitability of respiratory-related neurons. At the synaptic level, anesthetics/analgesics facilitate MOR and inhibitory GABA_A_ receptors while inhibiting excitatory NMDA and/or AMPA receptors. These effects on synaptic transmission may suppress respiratory drive and physical stimulation (*e.g*., CO_2_) to breathing.

Meanwhile, some general anesthetics, such as dexmedetomidine and ketamine, relatively preserve spontaneous breathing. These general anesthetics or sedatives produce respiratory depression until relatively high doses. Therefore, it is essential to understand the specific effects of various general anesthetics on respiratory control to develop novel anesthetics/analgesics with minimal respiratory depression and facilitate the safety of general anesthesia.

For pharmacological studies, although the overall regulatory effects of general anesthetics and analgesics on respiration are on multiple targets and complexes at the behavioral level, general anesthetics and analgesics can produce specific inhibitory and/or excitatory modulation in distinct respiratory-related nuclei at the cellular and molecular levels. Therefore, the modulation of anesthetics/analgesics on respiratory-related circuits may be used to change respiratory functions in future studies. For instance, volatile anesthetics, such as isoflurane and/or sevoflurane, can facilitate chemosensitive receptors, such as RTN, by enhancing the sodium background conductance and/or inhibiting the background potassium channel-like currents in phox2b neurons, so isoflurane and/or sevoflurane may be applied to maintain RTN chemosensitive activity in the brain stem slice recordings. Meanwhile, volatile anesthetics also inhibit astrocytic kir4.1 channels from modulating respiratory neurons indirectly, by which studies on astrocytes and rhythmogenic central pattern generators can be used. Although the effects of anesthetics are multiple, their specific effects on each ion channel and/or cellular subtype may be feasible for some studies restricted to region-specific brain nuclei at the molecular, cellular or circuit level. Since anesthetic and analgesic agents can affect various types of synaptic transmission and molecular targets in the respiratory system, they may also act as specific pharmacological tools to investigate respiratory control at the synaptic level.

In addition, it is possible to overexpress and/or knock down the expression level of molecular targets regulated by anesthetics and analgesics in certain specific nuclei and then explore the controlling effects of anesthetics and analgesics on their neuropharmacological actions other than respiration. For example, it is interesting and promising to use the anesthetics as tool drugs to investigate the mechanisms of anesthetics on consciousness and/or memory.

## Figures and Tables

**Fig. (1):**
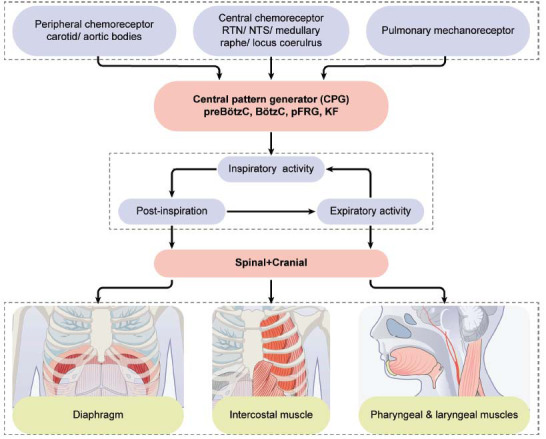
The mechanisms of the drive and control of respiration. Breathing movements depend on the coordinated activities of the neural system, which mainly originates from the central pattern generator, including pre-Bötzinger complex and Bötzinger complex, then send cranial and spinal motor outputs to respiratory muscles, including the diaphragm, intercostal muscle, and pharyngeal and laryngeal muscles. Meanwhile, the central pattern generators received inhibitory and excitatory inputs from the central and/or peripheral chemoreceptors and pulmonary mechanoreceptors. Ultimately, the rhythmically respiratory movement occurs, which comprises three phases: inspiration when inspiratory muscles contract, post-inspiration when inspiratory muscles progressively cease contraction, and active expiration when the expiratory muscles contract.

**Fig. (2):**
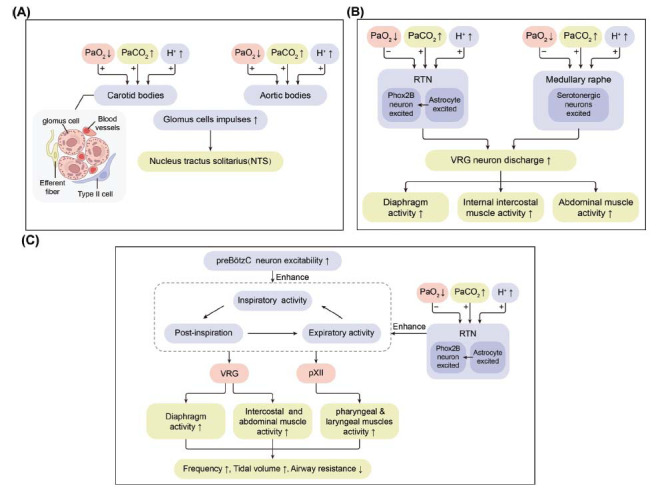
Modulations on the dynamics of brain regions and nuclei and how to change respiratory outputs. (**A**) Schematic view of peripheral chemoreflex microcircuits. The image shows peripheral chemoreceptors (carotid body and aortic body) and nucleus tractus solitarius are composed of peripheral chemoreflex microcircuits. The peripheral chemoreceptors are proposed as key elements in the detection of changes in the partial pressure of O_2_ (PO_2_), the partial pressure of CO_2_ (PCO_2_), and pH in arterial blood, and signal to the brainstem nucleus tractus solitarius. (**B**) Schematic view of central chemoreflex microcircuits. In the brainstem, chemosensory neurons and astrocytes in the retrotrapezoid nucleus (RTN) and/or medullary raphe detect and respond to fluctuations in CO_2_ levels and pH in the cerebrospinal fluid. These neurons project to the ventral respiratory group, including preBötzinger Complex (preBötC) and Bötzinger Complex, innervate pump and airway muscles. (**C**) The neurons of RTN directly project to the preBötzinger complex to modulate respiratory movement to maintain homeostasis. VRG = ventral respiratory group; preBötC = preBötzinger Complex, NTS = nucleus tractus solitarius.

**Table 1:** The effects of general anesthetics on respiratory parameters.

-	Intravenous Anesthetics					Volatile Anesthetics				
	**Ketamine**	**Dexmedet-omidine**	**Propofol**	**Etomidate**	**Thiopental**	**Isoflurane**	**Sevoflurane**	**Enflurane**	**Desflurane**	**Halothane**
Frequency	-[63, 66]	-[71-73]	+ (2.5 mg/kg bolus) [62] N/A (4 mg/kg slower administration) [66]	- (0.3 mg/kg) [67]	+[62, 67]	+ (1MAC) [4] - (>1MAC) [77]	+ (1MAC) [4] - (>1MAC) [77]	+ (1MAC) [4]	+ (1MAC) [4]	+ (1MAC) [4] - (>1MAC) [77]
Tidal volume	-[63, 64]	-[71-73]	-[62]	-	-[62]	- (1MAC) [4]	- (1MAC) [4]	- (1MAC) [4]	- (1MAC) [4]	- (1MAC) [4]
HCVR	-[63, 86, 87]	-[88]	-[62]	-[85]	-[62]	-[77]	-[77]	-	-[77]	N/A (0.5%) [92]
HVR	-	-	-[8, 9, 96]	-	-	-[90, 98]	-[90, 98]	-[90, 98]	-[90, 98]	-[97]
Upper airway patency	+[112, 117, 119]	-[118]	-[112, 117]	-	-[116]	-[120]	-[202]	-[124]	-	-[122]
Airway responses	-[126]	-[127]	-[125,126]	-[125]	+[125, 126]	-/+[128-130]	-[129,130]	-[131]	+[128, 132, 133]	-[129,130]

**Note**: “+”: Enhancement, “–”: Inhibition, “N/A”: No effect. HCVR: hypercapnic ventilatory response; HVR: hypoxic ventilatory response.

**Table 2:** Molecular targets of general anesthetics for respiratory modulation.

-	Intravenous Anesthetics					Volatile Anesthetics				
	**Ketamine**	**Dexmede-tomidine**	**Propofol**	**Etomidate**	**Thiopental**	**Isoflurane**	**Sevoflurane**	**Enflurane**	**Desflurane**	**Halothane**
Ca_v_	-	-	-[90]	-	-	-[211]	-	-	-	-
K_v_	-	-	-	-	-	-	-	-	-	-
Na_v_1.4	-	-	-[216]	-	-	-[216]	-	-	-	-
NMDAR	-[147]	-	-	-	-	-	-	-	-	-
APMAR	-	-	-	-	-	-	-	-	-	-
GABAR	-	-	+[96]	-	-	-	+[150]	-	-	-
nAChR	-	-	-[102]	-	-	-	-	-	-	-
μOR	+[63, 155]	-	-	-	-	-	N/A [154]	-	-	-
TASK-1	-	-	+[227]	-	-	+[228]	+[163]	-	-	+[163]
THIK-1	-	-	-	-	-	-[78]	-	-	-	-
Kir4.1/5.1	-	-	-	-	-	-[162]	-	-	-	-
NALCN	-	-	-[15]	-	-	+[15]	+[15]	-	-	-

**Note**: “+”: Enhancement, “–”: Inhibition, “N/A”: No effect. HCVR: hypercapnic ventilatory response; HVR: hypoxic ventilatory response.

**Table 3:** Potential stimulation targets for respiratory depression.

-	General Anesthetics			Opioids Analgesics		
preBötzC	-	-	-	5-HT4A receptors [224]	-	-
RTN	Kir4.1/5.1 [162]	THIK-1 [78]	NALCN [15]	-	-	-
Parabrachial nucleus	**-**	-	-	5-HT 2A receptors [222]	Cholecystokinin A receptors [222]	tachykinin receptor 1 [222]
CB	BKCa [234]	TASK-1 [106, 168, 169]	TASK-3 [234]	BKCa [228,234]	-	-

**Abbreviations**: preBötzC: preBötzinger complex; RTN: retrotrapezoid nucleus; CB: carotid body; BK_Ca_: Ca^2+^-dependent KCa1.1.

## LIST FOR ABBREVIATIONS

AMPA: Alpha-amino-3-hydroxy-5-methylisoxazole-4-propionate
BK_Ca_: Ca^2+^-dependent K_Ca_1.1
BötzC: Bötzinger Complex
CB: Carotid Body
GABA: γ-aminobutyric Acid
HCVR: Hypercapnic Ventilatory Response
HVR: Hypoxic Ventilatory Response
5-HT: Serotonin
I(h): Hyperpolarization-activated Cationic Current
K2P: Two-pore Domain Potassium Channel
KF nucleus: Kölliker-Fuse Nucleus
Kir: Inward Rectifier Potassium Channel
LC: Locus Coeruleus
MAC: Minimum Alveolar Concentration
MORs: μ-opioid receptors
nAChRs: Nicotinic Acetylcholine Receptors
NALCN: Leak Sodium Channel
NMDA: N-methyl-D-aspartate,
NTS: Nucleus Tractus Solitarius
OIRD: Opioid-induced Respiratory Depression
PBC: Parabrachial Complex
PetCO_2_: End-tidal PO_2_
PND: Phrenic Nerve Discharge
preBötzC: preBötzinger Complex
RBSNs: Respiratory Bulbospinal Neurons
RTN: Retrotrapezoid Nucleus
TASK: TWIK-related Acid-sensitive Potassium Channels
THIK-1: Tandem Pore Domain Halothane Inhibited Potassium Channel 1
VRG: Ventral Respiratory Group
